# Extrahepatic Manifestations and Autoantibodies in Patients with Hepatitis C Virus Infection

**DOI:** 10.1155/2012/871401

**Published:** 2012-09-05

**Authors:** Takashi Himoto, Tsutomu Masaki

**Affiliations:** ^1^Department of Gastroenterology and Neurology, Kagawa University School of Medicine, Kagawa, Japan; ^2^Department of Integrated Medicine, Kagawa University School of Medicine, 1750-1, Ikenobe, Miki-Cho, Kita-Gun, Kagawa 761-0793, Japan

## Abstract

Patients with chronic hepatitis C virus (HCV) infection frequently have many extrahepatic manifestations, as persistent HCV infection often triggers lymphoproliferative disorders and metabolic abnormalities. These manifestations primarily include autoimmune disorders such as cryoglobulinemia, Sjögren's syndrome, and autoimmune thyroid disorders. It has been well established that chronic HCV infection plays important roles in the production of non-organ-specific autoantibodies, including antinuclear antibodies and smooth muscle antibodies, and organ-specific autoantibodies such as thyroid autoantibodies. However, the clinical significance of autoantibodies associated with the extrahepatic manifestations caused by HCV infection has not been fully recognized. In this paper, we mainly focus on the relationship between extrahepatic manifestations and the emergence of autoantibodies in patients with HCV infection and discuss the clinical relevance of the autoantibodies in the extrahepatic disorders.

## 1. Introduction

Persistent hepatitis C virus (HCV) infection has been well characterized as having a preferential evolution which often evokes lymphoproliferative disorders [1] and metabolic abnormalities [2]. Therefore, patients with chronic HCV infection frequently develop extrahepatic manifestations [3–5]. Previous studies have revealed that 38–76% of patients with chronic HCV infection develop at least one extrahepatic manifestation [6–8]. These extrahepatic manifestations mainly include autoimmune disorders such as mixed cryoglobulinemia, Sjögren's syndrome, and thyroid autoimmune disorders. 

On the other hand, persistent HCV infection is responsible for the production of a variety of autoantibodies including non-organ-specific autoantibodies and organ-specific autoantibodies, as a virus-induced autoimmune phenomenon. The diversity of autoantibodies in the sera of patients with HCV-related chronic liver disease (CLD) [9–13] has been shown. Some autoantibodies in chronic HCV infection have biochemical, histological, or genetic characteristics, while other autoantibodies may predict the response to antiviral treatments, concomitant disorders, or prognosis in patients with HCV-related CLD [14]. 

Various mechanisms for the production of autoantibodies in patients with HCV-related CLD have been proposed. Molecular mimicry between a component of a virus and a “self” protein may account for the production of autoantibodies in chronic HCV infection [15]. A sequence homology between the HCV polyprotein and cytochrome p450 2D6 (CYP 2D6), which was identified as the antigenic target of antibodies to liver-kidney microsome type 1 (anti-LKM1), was previously reported [16]. The reactivity against the viral protein induces the production of anti-LKM1 in HCV-related CLD. Polyclonal B-cell activation by persistent HCV infection has been proposed as another mechanism for the production of autoantibodies [17]. B-cell proliferation seems to be essential for the development of autoimmune disorders including Sjögren's syndrome and mixed cryoglobulinemia (MC). Genetic predisposition is also strongly related to the presence of autoantibodies in chronic HCV infection [18]. The susceptibility to develop non-organ specific autoantibodies (NOSA) appears to be restricted to a specific human leukocyte antigen (HLA) in patients with HCV infection [19].

 The presence of NOSA including antinuclear antibodies (ANAs) and smooth muscle antibodies (SMAs) is associated with the severity of necroinflammation and fibrosis in the liver of patients with HCV-related CLD [20–24]. It is notable that the titers of these autoantibodies seem to be independent of HCV genotypes or loads of HCV-RNA [21–25]. The emergence of these autoantibodies did not affect antiviral treatments. [23]. However, we have to exclude concomitant autoimmune hepatitis (AIH) from patients with HCV infection seropositive for NOSA, because antiviral treatment occasionally exacerbates AIH in those patients [26].

The clinical significance of autoantibodies in the extrahepatic manifestations caused by HCV infection has been rarely discussed. This paper highlights the aspects of autoantibodies in extrahepatic manifestations by HCV infection and elucidates their clinical and therapeutic implications.

## 2. Extrahepatic Manifestations and Their Associated Autoantibodies

### 2.1. Cryoglobulinemia

Cryoglobulinemia is one of the most common extrahepatic diseases in patients with HCV infection and is detected in 19–54% of those patients [8, 27–30]. Cryoglobulins are immunochemically classified into three types according to the method by Brouet and his colleagues [31]. Type I cryoglobulins are composed of a monoclonal immunoglobulin and are often associated with hematological disease. Type II cryoglobulins are immune complexes consisting of polyclonal IgG with monoclonal rheumatoid factor (RF) activity, while type III cryoglobulins are characterized by polyclonal IgG with polyclonal RF. Therefore, type II and type III cryoglobulins are referred to mixed cryoglobulins. Persistent HCV infection is strongly associated with types II and III mixed cryoglobulinemia (MC) and occasionally associated with type I cryoglobulinemia. Cryoprecipitates contain HCV core proteins, IgG molecules with specific anticore activities, and IgM molecules with RF activities. C1q proteins and C1q binding activity were enriched in this immune complex [32], and were related to the wide expression of C1q receptor on the surface of blood cells and endothelial cells [33–35]. 

MC secondary to HCV infection often involves other organ systems in, for example, cutaneous manifestations, peripheral neuropathy, and glomerular disease [1, 36, 37]. There are interesting issues in the relationship between the emergence of cryoglobulin and more advanced hepatic fibrosis in patients with chronic HCV infection [27, 30, 38, 39]. However, all patients with HCV-related MC do not suffer from these involvements [8, 25, 30]. Overt vasculitis is observed in only 2-3% of patients with HCV-related MC [7, 40, 41]. The circumstances predisposing HCV-infected patients to develop these manifestations remain obscure.

It is noteworthy that cryoglobulins are usually found at low concentrations in patients with chronic HCV infection [25, 28, 29]. Patients with HCV-related cryoglogulinemic vasculitis had higher cryocrit levels than those without vasculitis [42]. Patients with cryoglobulinemic vasculitis had clinical characteristics of female-predominance, older age and longer disease duration [42]. The natural history and prognosis of cryoglobulinemic vasculitis was highly dependent on renal involvement or the severity of vasculitis lesion.

The precise mechanism by which HCV infection involves MC has not been well established. However, one hypothesis of a possible role played by HCV in polyclonal B-cell response has been proposed as follows: HCV has a strong affinity to the tetraspanin (CD81) ligand on the surface of B lymphocytes via the E2 protein (the second proportion of the HCV envelope) [43]. CD81 forms a costimulatory complex with CD19 and CD21 [44]. The ligation of CD81 on B cells results in the activation of this complex, which lowers the antigen threshold necessary for antibody production and eventually causes the formation of cryoglobulins [33]. The cryoglobulins initially produced are polyclonal IgG (type III MC), but as a dominat B-cell clone emerges, it may produce monoclonal immunoglobulins (type II MC) [9].

On the other hand, B-cell-activating factor (BAFF), a member of the tumor necrosis factor-alpha (TNF-*α*) family that plays crucial roles in B-cell differentiation, survival, and immunoglobulin secretion, is considered to be associated with the development of autoimmune disorders [45]. The elevation of serum BAFF levels was observed in patients with HCV-related lymphoproliferative disorders [46, 47] which represents a link between infection and autoimmunity. Quantitative decrease in regulatory T cells may be involved in patients with HCV-related MC [48]. 

Serological hallmarks reflecting autoimmunity in patients with HCV-related MC have been fully recognized.  Table 1 summarizes the clinical characteristics of autoantibodies in patients with HCV-related MC. Non-organ-specific autoantibodies including ANA and SMA have been observed in 12–65% [8, 29, 36, 49–52] and 33–37% [8, 53] of patients with HCV-related MC, respectively. It is noteworthy that the immunofluorescence pattern of ANA on HEp-2 cells in HCV-related MC was speckled [47, 51]. Rheumatoid factor (RF), which recognizes the Fc portion of IgG molecules as their antigens, often appears in sera of patients with HCV-related MC, at frequencies of 14 to 99% of those patients [30, 36, 49, 52, 54, 55]. However, the titers of ANA and RF in sera of HCV-related MC were less than 1 : 80 and 50 UI/mL, respectively [51], which appeared to be low. Antineutrophil cytoplasmic antibodies (ANCAs), which are divided into two groups by immunofluorescence patterns: pANCA and cANCA [56], are also present in the sera of patients with HCV-related MC [8, 57, 58]. However, the occurrence of these autoantibodies was not necessarily related to HCV-related cryoglobulinemic vasculitis [57].

Some types of autoantibodies are available for predictive markers of HCV-related MC. The presence of circulating autoantibodies to C-reactive protein antibodies (anti-CRP), which are directed against monomeric CRP [64], was dependent on HCV-related MC [59, 60]. In addition, antibodies to C1q, which is closely associated with immune complex diseases including hypocomplementaemic urticarial vasculitis and systemic lupus erythematosus (SLE) [65], was detected in 38% of patients with chronic HCV infection [66], implying that the emergence of anti-C1q represented concurrent cryoglobulinemic vasculitis. However, Saadoun and his colleagues revealed that anti-C1q was not associated with cryoglobulinemic vasculitis, but indicated the susceptibility to type III MC in those patients [61].

Other types of autoantibodies are closely linked to the organ involvements in patients with HCV-related MC. Positivity for RF seems to reflect the development of cutaneous vasculitis in patients with HCV-related MC. Karisberg and his colleagues demonstrated that all HCV-related MC patients with cutaneous vasculitis had RF and liver involvement [67]. The detection of Type II cryoglobulins containing RF (type II-RF) appeared to monitor cryoglobulinemic vasculitis in those patients [68]. IgM*κ* RF was strongly associated with membranoproliferative glomerulonephritis (MPGN) in type II MC [69]. On the other hand, a recent study reported by Knight and his colleagues elucidated that the monoclocal RFs that bear the WA cross-idiotype are responsible for vasculitis in patients with HCV-related MC [70]. 

Antiendothelial cell autoantibodies (AECAs) were recently identified as a serum parameter for an autoantibody against a variety of antigen determinations on endothelial cells and their titers represented the activity of vasculitis [71]. Cacoub and his colleague revealed that AECA was present in 50% of patients with HCV-related MC and that seropositivity for AECA was associated with the prevalence of vasculitis and serum cryoglobulin levels in those patients [62]. However, the authors did not describe the correlation between the AECA titers and the activity of vasculitis. Elevated soluble vascular cell adhesion molecule-1 (VCAM-1) was likely to contribute to the involvement of vasculitis in HCV-related MC. Therefore, AECA-induced activation of endothelial cells may initiate an upregulation in the expression of endothelial adhesion molecule. 

The analysis of antineuroual antibodies [72] including anti-ganglioside GM1 and anti-sulfatide antibodies was performed using the sera of HCV-related MC [63]. The association between those titers and the involvement of the peripheral nervous system was apparent in patients with HCV-related MC.

Genetic susceptibilities may be related to the development of MC in patients with chronic HCV infection. HLA DRB1*11 (DR11) was found to predict cryoglobulinemic vasculitis in patients with HCV infection, whereas HLA DR7 seemed to protect from the development of type II MC [73, 74]. 

### 2.2. Sjögren's Syndrome

Sjögren's syndrome (SS) is another common extrahepatic manifestation caused by HCV infection. 6–26% of patients with chronic HCV infection complain of sicca syndrome (xerostomia and/or xerophthalmia) [6–8, 33, 75, 76]. The pathogenesis of HCV-associated SS is not well established. The virus is unlikely to have a direct effect, because HCV has not been proven in glandular tissue [77, 78]. The proposed mechanism includes cross-reactivity between the HCV envelope and host salivary tissue or HCV envelope-mediated salivary glands. Koike and his colleagues elucidated the resemblance of salivary lesions from a transgenic model overexpressing the HCV envelope protein [79].

SS secondary to HCV infection can be distinguished, to some extent, from primary SS by analyzing several types of autoantibodies (Table 2). The seropositivities for antibodies to SS-A/Ro and to SS-B/La are much lower in HCV-associated SS than those in primary SS patients [75, 80]. The prevalences of cryoglobulin and RF were higher in the sera of patients with HCV-associated SS than in those of primary SS patients [75, 80, 81]. It is of interest that the coexistence of cryoglobulinemia in HCV-related SS may favor the development of lymphoproliferative diseases including B-cell NHL [82]. There has been recent discussions on the relevance of antibodies to alpha-fodrin in patients with HCV-related SS seronegative for antibodies to SS-A/Ro [83].

Apart from the antibody status, HCV-related SS has several clinical characteristics distinct from primary SS. Biochemical analysis revealed a higher frequency of hypocomplementemia in HCV-SS than primary SS [80, 84]. The imbalance of Th1/Th2, namely, poor Th1 response and enhanced Th2 response, was apparent in HCV-related SS [80]. A recent study revealed that the detection of monoclonal gammopathy (IgM*κ*) might help to distinguish HCV-related SS from primary SS [85]. De Vita and his colleagues documented higher prevalence of monoclonal gammopathy in HCV-related SS than that in primary SS [86]. Histological examination of the salivary gland in HCV-related SS showed milder pericapillary and nonpericanalary lymphocytic infiltration than primary SS [87]. The prevalence of liver involvement was far higher in HCV-related SS than in primary SS [84].

Patients with the HLA haplotype of HLA DQB1*02 had a susceptibility to the development of HCV-related SS [88]. On the other hand, the prevalence of HLA DR 3 [89], a haplotype specific to primary SS, was far lower in patients with HCV-related SS [75]. 

### 2.3. Autoimmune Thyroid Disease

Thyroid disorders are common in patients with chronic HCV infection. Approximately 10–25% of patients with persistent HCV infection have thyroid autoantibodies, including thyroid microsome autoantibodies (TMAs), thyroglobulin autoantibodies (TGAs), and antibodies to thyroid peroxidase autoantibodies (anti-TPO), regardless of the liver involvement severity [90–92]. TMAs are frequently useful to detect latent autoimmune thyroiditis in patients with CH-C prior to antiviral treatment [91]. The presence of TMAs also may predict thyroid dysfunction including hyperthyroidism and hypothyroidism [93]. Therefore, the detection of these thyroid autoantibodies is considered useful for the clinical diagnosis of concurrent autoimmune thyroid diseases in patients with HCV infection. However, HCV-associated thyroid disorders cannot be distinguished from primary thyroid disorder by the existence of these thyroid autoantibodies. The possible role of HCV in the development of thyroid disorders has not been fully understood.

HCV-related thyroid disorders include Graves' disease and Hashimoto's thyroiditis induced by interferon (IFN) treatment [94, 95]. The discovery of anti-TPO at base-line may be regarded as a predictive factor for IFN-induced thyroid autoimmunity in patients with CH-C. A strong correlation between thyroid disorders and the presence of anti-LKM1 at base-line in patients with chronic hepatitis C was also observed [96, 97]. HCV poly protein, CYP2D6 and thyroperoxidase are likely to share epitopes encoding homologous amino acid sequences [97]. Therefore, seropositivity for anti-LKM1 is a susceptibity factor for IFN-induced autoimmune thyroid disorders in patients with HCV-related CLD. 

### 2.4. Lichen Planus

Lichen planus is well known to be a skin lesion associated with persistent HCV infection, although the pathogenesis remains uncertain [98]. Approximately 1–6% of patients with chronic HCV infection have been estimated to be afflicted with oral lichen planus [7, 8, 49]. The existence of concurrent lichen planus was associated with chronic active hepatitis [99], suggesting that the chronic HCV infection alone did not cause lichen planus. However, the severity of hepatic fibrosis and necroinflammation was independent of the severity of lymphocytic infiltration in the oral lichen planus [100].

Nagao and her colleagues revealed that the emergence of antibodies to cardiolipin (anti-CL), the hallmark of antiphospholipid syndrome [101], might reflect concurrent oral lichen planus in patients with chronic HCV infection [102], although the association seems to be controversial [103]. Surprisingly, these patients with anti-CL did not fulfill the criteria for antiphospholipid syndrome [102]. The emergence of TMA may be associated with oral lichen planus secondary to HCV infection [104], although the putative mechanism was not described in detail. Clinical relevance of antibodies to epithelial components in HCV-associated oral lichen planus was also reported [105]. To the contrary, Carrozzo and his colleagues elucidated no relationship between the emergence of autoantibodies including ANA, SMA, parietal cell antibodies, and anti-epithelial antibodies and concomitant lichen planus in patients with HCV infection [106]. Another study revealed that cryoglobulin-positive patients with CH-C had higher prevalence of lichen planus than cryoglobulin-negative patients [6].

### 2.5. CREST Syndrome

CREST syndrome is a rare concurrent autoimmune disease in patients with persistent HCV infection [3]. We previously elucidated that approximately 1% of patients with HCV-related CLD have anticentromere antibodies (ACAs) [107], the serological hallmark of CREST syndrome [108]. However, all patients seropositive for ACA did not have symptoms of CREST syndrome [107], like the cases of primary biliary cirrhosis (PBC) patients with ACA [109]. The putative role of persistent HCV infection in the production of ACA remains uncertain, although the molecular mimicry between the HCV core antigen and CENP-A [110], one of the major centromere proteins [111], has been shown.

### 2.6. IFN-Induced Autoimmunity

Treatment with IFN-*α*, an antiviral drug, can precipitate or exacerbate autoimmune endocrine diseases as well as autoimmune thyroid disorders in patients with CH-C [101]. Treatment with IFN-*α* resulted in the upregulation of major histocompatibility complex (MHC) class I expression on thyroid epithelial cells and switching the immune response to the Th1 pattern and subsequent cytokine release (IFN-*γ* and interleukin-2) [93–95, 112]. The thyroid was the organ most susceptible to treatment with IFN-*α* in patients with CH-C [113].

The association of the IFN treatment with the development of type 1 diabetes mellitus (DM) has been shown in patients with CH-C patients. Of all CH-C patients, 1.4–2.8% had antibodies to glutamic acid decarboxylase (anti-GAD), the serological hallmark of type 1 DM [114], prior to the treatment with IFN-*α* [115, 116]. Most CH-C patients with anti-GAD developed type 1 DM by the IFN therapy. The onset of type 1 DM was closely restricted to the genetic susceptibility demonstrated by the presence of the HLA DRB1-DQB1 haplotype in those patients [117].

Antibodies to 21-hydroxylase were observed in 4.8% of patients with CH-C receiving IFN treatment. However, the presence of this autoantibody was independent of adrenal failure [118].

The existence of non-organ-specific autoantibodies including ANA at end of the treatment or the increase in titers of SMA can predict unfavorable outcomes of antiviral treatments in patients with CH-C [119].

It is of interest that Covini and his colleagues identified a novel autoantigen, which appeared like distinct rods and rings (RRs) in the cytoplasm of HEp-2 cells, in patients with CH-C under treatment with pegylated IFN-*α* and ribavirin [120].

Genetic backgrounds may trigger the development of IFN-induced thyroid disorders. There was a close correlation between HLA A2 and IFN-induced autoimmune thyroiditis in Japanese patients with CH-C [121]. Another study revealed the association of DRB1*11 with IFN-induced autoimmune thyroiditis in a Caucasian population [122]. 

### 2.7. Malignant Transformation

An oncogenic role in chronic HCV infection has been widely shown by the development of hepatocellular carcinoma (HCC) and B-cell NHL [123]. The close relationship between carcinogenesis and autoimmunity has been also well recognized.

A previous issue revealed that the minority of patients with HCV-related MC (5–10%) develop a frank malignant lymphoma in long-term follow-up [124]. The clonal B-cell expansion by HCV infection may account for the malignant transformation in patients with HCV-related MC [125]. The *t* (14 : 18) translocation with overexpression of bcl-2 anti-apoptotic protein [126] in B cells leading to extension of B-cell survival [127, 128] and the subsequent mutations of oncogenes including c-myc and p53 [129, 130] seemed to play essential roles in the development of B-cell NHL.

A strong linkage between HCV-related SS and B-cell NHL has been widely documented. Ramos-Casals and his colleagues recently demonstrated that patients with HCV-related SS who developed NHL had the immunological features of higher prevalence of RF and type II MC than those with HCV-related SS without NHL [82].

Monoclonal gammopathy of undetermined significance (MGUS) has been recognized as another lymphoproliferative disorder in HCV-related MC [131]. The monoclonal gammopathies of IgG*κ* and IgM*κ* are frequently observed in HCV-related MC [132]. The monoclonal gammopathy in those patients should be monitored to exclude the possibility of an evolution to multiple myeloma [133].

Raedle and his colleagues revealed that three of 7 (43%) patients with HCV-related HCC had autoantibodies to p53 [134], one of the tumor-associated antigens [135], while none of the patients with HCV-related CLD did, suggesting that HCV-induced carcinogenesis resulted in the production of these autoantibodies. On the other hand, the prevalence of autoantibodies to survivin [136], a protein which belongs to the inhibitor-of-apoptosis protein (IAP) family [137], was higher in patients with HCV-related HCC than in those with HBV-related HCC. We previously reported 8 of 86 (9%) patients with HCC had autoantibodies to tumor-associated antigens including p53, *i*nsulin-like growth factor II *m*RNA-binding *p*roteins (IMPs), c-myc, and survivin [138, 139]. It is of note that seven of 8 HCC patients with those antibodies were HCV related [138, 139].

A few types of autoantibodies frequently observed in autoimmune diseases are generally present in sera of patients with HCC. There is an interesting issue on the relationship between autoantibodies to Golgi, which are usually seen in the patients with rheumatoid arthritis and SLE [140] and HCV-related HCC [141]. Our present report revealed that patients with HCV-related CLD seropositive for ACA frequently developed HCC [107]. Antibodies to CENP-B, the most common target antigen of ACA, were regarded as a potential biomarker for small-cell lung cancer [142].

### 2.8. Autoimmune Cytopenia

As another extrahepatic manifestation, reports of HCV-related autoimmune cytopenia including autoimmune hemolytic anemia (AHA), autoimmune thrombocytopenia, and autoimmune neutropenia are recently increasing in number [143]. It is of interest that patients with HCV-related AHA are often associated with the emergence of ANA and MC [143]. Patients with HCV-related autoimmune thrombocytopenic purpura also had more immunological markers [144].

On the other hand, 66–88% of patients with chronic HCV infection and thrombocytopenia had antiplatelet antibodies [145, 146], although the development of antiplatelets is not associated with thrombocytopenia. The most common target antigen of anti-platelet antibodies is glycoprotein (GP) IIb/IIIa [146].

### 2.9. Atherosclerosis

Atherosclerosis has been described as a metabolic abnormality caused by HCV infection [147]. Autoantibodies to oxidized low-density lipoprotein (anti-ox-LDLs) have been identified as a serological hallmark of atherosclerosis [148]. We recently revealed that anti-ox-LDLs were associated with the severity of hepatic steatosis in patients with CH-C [149]. Further examination will be required whether anti-ox-LDL become a biomarker for atherosclerosis in patients with chronic HCV infection or not.

## Figures and Tables

**Table 1:** Autoantibodies detected in sera of patients with HCV-related MC.

Autoantibodies	Frequency (%)	Clinical significance	References
ANA	12–65%	Low titer	[8, 30, 37, 49–52]
		Speckled pattern on HEp-2 cells	
SMA	33–37%	Low titer	[8, 53]
RF	14–99%	Low titer	[30, 37, 49, 52, 54, 55]
ANCA (pANCA, cANCA)	4–26%	No association with vasculitis	[57, 58]
Anti-CRP	75%	Predictive marker of cryoglobulinemia	[59, 60]
Anti-C1q	39%	Predictive marker of type III cryoglobulinemia	[61]
AECA	50%	Predictive marker of vasculitis	[62]
Anti-GM1 ganglioside antisulfatide	52%	Predictive marker of peripheral neuropathy	[63]

**Table 2:** Comparisons of immunological and histological findings between primary SS and HCV-related SS.

Variable	Primary SS	HCV-related SS
Autoantibodies	High frequency of ANA, anti-Ro, and anti-La	Low frequency of ANA, anti-Ro, and anti-La
Cryoglobulin	Rare	Common
Hypocomplementemia	Rare	Common
Lymphocytic capillaritis	Moderate to severe	Mild to moderate
Th1/Th2 balance	Th1 predominant	Th2 predominant
Monoclonal gammopathy	Low	High
Association of HLA DR-3	High	Low

## Abbreviations

ACA: Anticentromere antibodies
AECA: Antibodies to endothelial cells
AHA: Autoimmune hemolytic anemia
AIH: Autoimmune hepatitis
anti-CRP: Antibodies to C-reactive protein
anti-GAD: Antibodies to glutamic acid decarboxylase
AMA: Antimitochondrial antibodies
ANA: Antinuclear antibody
anti-CL: Antibodies to cardiolipin
anti-IMPs: Antibodies to insulin-like growth factor II mRNA-binding proteins
anti-LKM1: Antibodies to liver kidney microsome type 1
anti-ox-LDL: Antibodies to oxidized low-density lipoprotein
anti-TPO: Antibodies to thyroid peroxidase
BAFF: B-lymphocyte activating factor
c-ANCA: Antineutrophil cytoplasmic antibody with cytoplasmic pattern,
CH-C: Chronic hepatitis C
CLD: Chronic liver disease
CYP2D6: Cytochrome p450 2D6
DM: Diabetes mellitus
HCC: Hepatocellular carcinoma
HLA: Human leukocyte antigens
IAP: Inhibitor of apoptotic proteins
IFN: Interferon
MHC: Major histocompatibility complex
NHL: Non-Hodgkin's lymphoma
NOSA: Non-organ specific autoantibodies
pANCA: ANCA with perinuclear pattern
MC: Mixed cryoglobulin
PBC: Primary biliary cirrhosis
RF: Rheumatoid factor
SLE: Systemic lupus erythematosus
SMA: Smooth muscle antibody
TGA: Thyroglobulin antibody
TMA: Thyroid microsomal antibody
VCAM-1: Vascular cell adhesion molecule-1.
